# Seventh Annual Session of the Maryland State Dental Association

**Published:** 1890-09

**Authors:** 


(To be continued.)

---

### SEVENTH ANNUAL SESSION OF THE MARYLAND STATE DENTAL ASSOCIATION.

(Continued from page 499.)

### DISCUSSION ON OPERATIVE DENTISTRY.

*Dr. T. S. Waters*, of Baltimore.—I am an adherent of both soft and cohesive gold, and I claim to exercise my judgment in using either. I think that, as has been remarked, we should all be conservative in our practice. I suggest that the consideration as to the material with which a tooth is filled is not the only one to be borne in mind. If the cavities have not been properly shaped and if the operation has not been performed with judgment, it matters not what the substance is that is made use of. Many failures have resulted from ignoring this fact as well as because of the selection of a certain kind of material for filling.

Another thing I have noticed is that, as practitioners, we do not pay that attention to the constitutional conditions of our patients which should be given them. My custom is to refer patients to their physicians, if they have a family physician. I am not practising medicine, and I have very frequently made suggestions to the physician, in a particular case, if I happened to be acquainted with him. As a result of this, I have found that the patients have been much benefited by the medical treatment they received, and that this treatment has very materially aided in the success of the dental operation which I subsequently performed. I have in my mind at this time the case of a lady who was in a very depressed condition. Her nervous system had run down and she was verging on nervous prostration. When she came to me the enamel of two central incisors had been very much corroded and the teeth were extremely sensitive. I asked her if she had an attending physician in the city, and she replied that she had not. I then prescribed a certain line of treatment. This was about two weeks ago. She returned to me on, I think, the day before the present meeting of this society, at which time she had an appointment with me to have her teeth filled. The condition of her nervous system had meanwhile much improved and, as a consequence of this improvement, the teeth were less sensitive. I prescribed lacto-phosphate before meals, a malt extract with her meals, and Fowler's solution of arsenic after meals. I then dismissed her with an injunction to return in eight or ten days, at the end of which time I hoped to be able to fill her teeth. I mention this as one instance of many of a like character. I could detail a number of cases in my practice in which the success of my operation has been promoted by the patient subjecting himself or herself to a course of treatment under the direction of a physician preliminary to an operation on the teeth. This medical treatment has aided me very materially in the work of preserving the teeth.

Dr. Harris has alluded to plastic fillings, and I want to say in regard to them that, in this anæmic, debilitated condition of the patient, a plastic filling is the thing to use as a temporary filling, which will serve the patient until the teeth have returned to their normal condition. The teeth may then be filled with gold or whatever substance the operator may deem advisable. Doubtless other gentlemen present have noticed the constitutional conditions of which I have spoken and may feel disposed to explain them more intelligibly than I have been able to do.

With respect to the comparison which has been made between cohesive and non-cohesive gold, gentlemen have stated that if they were obliged to give up either, they would prefer to abandon cohesive gold. I have only to say that, at the time of my graduation in dentistry, I had knowledge only of the non-cohesive form, but, since then, I have learned to use gold in all forms; and if compelled to make a choice between the two, I would give up non-cohesive foil. My reason for this is that I am an admirer of nature; and as the Divine Being has placed in the mouth teeth approximating each other, I am opposed to cutting away and leaving V-shaped spaces between the teeth, in which the food may impinge upon the gum and render it sore and inflamed. Nature has not intended that these V-shaped spaces should appear.

*Dr. R. Finley Hunt*, of Washington.—Not having heard the paper on Operative Dentistry, of course I am not so well prepared to discuss its contents as are the gentlemen who had the opportunity to listen to it; but the tendency of the discussion so far as it has continued since I have been present leads me to say one or two words in response to the kind invitation of the society.

A point was mentioned in the discussion which took place this morning with reference to the liability of teeth, particularly the bicuspids, to decay at the cervical wall after having been filled. I do not think that exemption from that liability is dependent so much on the material that is introduced into the cavity as it is upon the character of the permanent preparation of the tooth. I will probably be able to illustrate my meaning by mentioning the two points to which attention should be specially directed in the preparation of the cavity. We know that, in these cases, the cervical wall is the most difficult portion of the cavity to reach; and on that account it is frequently improperly prepared. I suggest, by the way, that it is a good rule for an operator to devote his closest attention in the preparation of a cavity to all of the most remote and inaccessible portions of that cavity. In the preparation of a cavity on the marginal surface of a bicuspid we may give as much attention as we please to the wall of the cavity, we may prepare the cervical wall by removing all the decay and softened portion of the tissue; but in almost every case an examination will show us that not only is the hard tissue in the cavity, but that the surface of the tooth above the cavity is affected. I speak now of superior bicuspids. If we fail to remove that softened portion of the tissue, and there is consequently a failure to resist the action of decay, caries will commence outside of the cavity, on the surface of the neck of the tooth. At the same time there is no doubt in my mind that the contact of amalgam with these softer dental tissues, the dentine and cementum, has a tendency to preserve that tissue. My experience has been that the material introduced becomes changed by chemical action upon certain metals entering into the composition of the alloy.

So much for amalgam and so much for the preparation of the cavity and the materials that are introduced. But, as suggested by Dr. Waters, even with the most perfect preparation of the cavity and the most thorough, careful, and successful introduction of the materials with which to fill it, if the systemic or constitutional condition of the patient is of a certain character, that tooth will afterwards decay. Unless you institute treatment so as to bring the system into a condition in which the hard tissues are rendered more dense and more capable of resisting the decay, it cannot be stopped. We all know that, after filling a tooth on one surface, the patient has come back in a short time with the filling good, but the tooth decayed in another part which had previously exhibited no symptoms of decay. Therefore I agree with Dr. Waters that our attention should be directed more closely than it has been to the systemic treatment of our patients.

I am reminded of a case occurring in my practice, that of a young girl some fifteen or sixteen years old, the daughter of an eminent physician of Georgetown. I found that her systemic condition was such that the acidity of the secretions of the mouth was neutralizing the salts of the tooth, and that, if this was not changed, it would be utterly useless for me to fill her tooth. This was reported to her father. He paid me a visit and examined me for an hour and a half as to my reasons for the statement I had made. It was an hour and a half of great anxiety to me, because I felt that I was on trial, or that, as representing the profession, my profession was on trial before an eminent physician. I satisfied him, however, and he placed his daughter under a course of treatment. She returned to visit me occasionally, and at the end of two or three months her condition was changed. I then set to work to fill those teeth which required filling; I used soft foil. Six years afterwards she visited me, and I found every one of the fillings in good condition and every other tooth intact. In my opinion, if this course of treatment had not been adopted, her teeth, at the end of six years, would have been almost a wreck. What I have said will serve to show the importance of paying attention to the systemic condition.

As Dr. Waters has said, we do not give sufficient attention to hygienic conditions. If every practitioner of dentistry would give more thought in that direction I think that as much, if not more, good would be done. Not only should our attention be given to patients who come to us with decayed teeth, but we should endeavor to bring about, through parents, an improvement in the systemic condition of the children.

Now let me say a word as to phosphate and chloride fillings. When cement fillings were first introduced, it was the boast of those who prepared them that they would withstand the action of the strongest acid. That boast was a well-founded one. As a rule, acid does not affect the condition of oxyphosphate and oxychloride fillings. They are affected by a different chemical agent. I may say that I have pursued a line of investigation on this question for a long time, and have consulted some of the ablest chemists in Washington on the subject. I became satisfied that the destruction of cement fillings was due to the presence of an alkali, and I consulted these eminent chemists in order to ascertain by what means, in their knowledge, we could determine the character of these secretions or exudations of the gum which gave rise to this alkali. They expressed their inability to give the information unless the secretions or exudations were collected in sufficient quantities. Of course it was very difficult to collect them. They said it would be almost impossible under the existing circumstances for them to ascertain what I desired to know. By the way, Mr. President, it has been my opinion, and one which I expressed many years ago, that erosion of the teeth, which is sometimes called abrasion, is produced not by the action of the tooth-brush, but by the presence of an alkaloid in its natural state, which is caused by an abnormal condition of the gum. We know as a fact that if we take an oxychloride or an oxyphosphate filling and drop it into ammonia, it is very rapidly disintegrated and destroyed. We may drop some of that same filling into an acid of any strength, and we will find that it is not affected. Now we find that those oxychloride and oxyphosphate fillings very often, and in a majority of instances, where they fail at all, fail at or near the cervical wall of the cavity, where the exudation from the gum comes in contact with the filling. I have often found the plastic or oxyphosphate fillings to be perfectly sound in every part except at the cervical wall. At that point a deep cavity in the filling would be found. This showed that the erosion had acted on that and penetrated to the depth of the cavity.

In support of that view, I may add (for I think it is well that we should mention all our reasons in a matter of this kind) that in the course of my investigation in past years I found, in almost every instance in which this erosion was taking place, that the paper test showed the actual character of the secretions of the gum in the neighborhood of the erosion, while in other parts of the mouth the reaction would be the reverse. So that I think that the failure of fillings of the material referred to is due, in a majority of cases, to the peculiar condition of the secretions of the mouth destructive to the structure of the fillings.

*Dr. Samuel J. Cockrell*, of Washington.—Not having been present when the essay was read, I am not informed as to the exact point at issue before the society, but I take it for granted that you are discussing what is the best material for the preservation of the teeth. The first question, then, is, Can teeth be permanently preserved by the use of gold or any other material? Gold was the material to which the attention of men of my age was called before we knew of any other substance. Dr. Waters has remarked that he was taught to fill teeth with non-cohesive foil only; and his statement will apply to many of the gentlemen now present. The subject is a very interesting one. That teeth can be saved by being filled with gold is a fact, and I think that persons who reach the age which has been reached by Dr. Hunt and myself and who have had the opportunity of seeing and learning what we have, will set it down as an undeniable fact that teeth can by this means be permanently preserved. Only a few days ago, when Dr. Magill came into my office, I had occasion to refer to certain teeth that were filled ten or fifteen years ago and which were now perfectly sound and good, though I regretted that I was unable to show the teeth to him. Facts such as these demonstrate that there is a material by the use of which teeth may be saved.

In more recent days we come to the work of our dear old friend, now on the other side of the river, who filled teeth with the same non-cohesive gold and saved them. I have among my patients at this time one whose teeth were filled by Dr. Arthur—it must have been thirty-five or forty years ago—with non-cohesive foil, and whose teeth are perfect to this day. Dr. Arthur then stopped using non-cohesive foil; and although many others have claimed the credit, Dr. Arthur was really the first to make use of that form of gold which is now termed cohesive or annealed gold. I had occasion, a few days ago, to refer to Dr. Arthur's work on the subject, entitled "Arthur's Annealed Gold," which he had kindly sent me. I have seen many operations from Dr. Arthur's hands with the same gold. He believed it to be a great improvement, but the results he obtained did not prove that it was equal to the other, for, having seen many of the operations, I can say that failures frequently occurred.

It is, however, only by reaching out for facts in a practical way that we can determine the actual worth of new materials and methods and thereby make an advance in our specialty. We know how many years of constant application are required to enable a man to educate himself in dentistry. Proficiency in our specialty cannot be acquired in a day. For the average man, at least thirty years of hard study and work are necessary to qualify him for the practice of his profession. In saying this I do not overlook the fact that there are exceptional instances in which men with a special aptitude for the profession may accomplish great results in a shorter length of time. But I repeat, that to qualify yourselves as dentists requires time, and I think that the erroneous idea which seems to have prevailed on this point has been unfortunate for the profession and has retarded its progress. I do not mean to say that some men may not acquire proficiency more rapidly than others; on the contrary, I realize that one man can accomplish greater results with a few excavators and a few pluggers, say half a dozen of each kind, than can be accomplished by less skilful operators with the very best set of instruments.

In coming to the city of Baltimore my heart has been filled to overflowing, for I do not forget that this place is the Bethlehem of dentistry; that to Chapin A. Harris we probably owe more than to any other man of the profession.

I may state here that Dr. Harris filled my teeth certainly not later than 1852, it may have been in 1851. The fillings have never been touched from that day to this; and, for an old man, the teeth are good now. Some twelve or fifteen of them are standing perfect. We have heard much about the materials for filling, but is there anything that will stand usage for these many years in comparison with the non-cohesive gold in the fillings in my mouth, which has during all this time preserved my teeth free from decay? That is the question to be considered, and it is one which all of you, gentlemen, have to ask yourselves.

You must use that material which is of the greatest benefit to your profession, to your patients, and to yourself. We understand the difficulty of filling teeth down at the cervical wall. If you can fill teeth there of the molar and bicuspid class, I think you will succeed anywhere and everywhere. That material which will do this the best, I claim, is the non-cohesive foil, or what is now termed the old-fashioned foil.

*Dr. Price.*—I have been using Steurer's foil for about a year, but not for a sufficient length of time to thoroughly test it. I may say that the more I use it the more I am pleased with the results I obtain. I think it is certainly one of the best forms of gold we have ever had prepared for our use. I am of the opinion we can press it more closely to the walls of the tooth than we can the others. It is especially fitted to work on frail teeth, and is also certainly well adapted for the labial walls of the teeth and in cases pertaining to that kind of work. But, as I have said, I have not yet used it for a sufficient length of time to enable me to ascertain how long the fillings are going to stand. As far as I have observed, it has done well, and fillings which I inserted a year ago are now just as perfect as they were on the day I put them in.

*Dr. A. J. Volck.*—I have a word to say, but I will say at the outset that it has no reference to the skill which an operator may have in preparing a cavity and putting in his gold, and it is irrespective of the condition of the cavity. I want to speak only of the properties of the various kinds of gold. I use principally non-cohesive gold; but when I use cohesive gold (and I use it freely in many cases), the great object with me is to make that foil as solid as it can possibly be made. In the very nature of the gold, by heating it and making it cohesive we make an absolutely solid mass of gold. I know this to be the fact, because I have taken some of those gold fillings and rolled them out and found that there was no failure of continuity in that material. Now the question with me has always been this: if we have a large gold filling in a tooth the walls of which are comparatively weak (and when the fillings are large the walls are generally more or less weak), and if we put in a material which perfectly fits that cavity, but a material which contracts and expands with the changes of temperature in the mouth, there must be a gradual destruction of that tooth. Those changes of temperature are very great, because people often drink a hot liquid of a temperature of 150° F. and follow it with a drink of ice-water, making a change in the temperature of the mouth of, perhaps, 112° F. in a single moment. When these changes are going on there must be either a breaking down of the walls or a still greater contraction of the gold, and consequent leakage.

Dr. Harris mentioned Dr. Webb's fillings, and I desire to say that Dr. Webb was unquestionably one of the most persistent users of cohesive foil that this country ever had. I know a gentleman now residing in this city who was formerly a superintendent in a nail and spike factory in Dr. Webb's town, and who has several of those large fillings, every one of which leak. I believe that the continual expansion and contraction of the gold is the cause of this, as it must necessarily in the end either wear out the walls of the cavity or destroy the filling by expansion and raising the surface.

I know, and the proof can readily be produced, that non-cohesive gold does not act in that way; that we can depend upon our ability to make a non-cohesive gold filling which does not leak and which lasts for many years. I know also the fact that in using cohesive foil we destroy many more teeth than when using non-cohesive foil. We should adapt ourselves to the two under certain conditions; but when we use cohesive foil we are obliged to cut away portions of the tooth that might otherwise be preserved.

Concerning the point as to the contraction and expansion of gold, I have only to call the attention of the profession to the fact that experiments can very easily be made to show that this is bound to injure the teeth in the end. When the fillings are small it may not have so much effect, but in some large fillings, which are to a large extent surrounded by the tooth-structure, failure is sure to follow, and I have never seen one such filling that did not break down.

*Dr. T. S. Waters.*—I desire to ask Dr. Volck one question. He knows that there are large amalgam fillings that are preserving the teeth and have been preserving them for years, and that these are conductors of heat and cold and subject to the same influences to which the large gold fillings spoken of are exposed. How can the doctor account for this?

*Dr. Volck.*—I do not believe that any amalgam filling was ever made that did not shrink some and leave some little space. Now, I have seen amalgam fillings tested in tubes. That is all very well, but it is one thing to put amalgam in a little tube and another thing to put it in the teeth.

*Dr. Waters.*—It has been proved that an amalgam filling does not leak, by testing in the mouth.

*Dr. Volck.*—I have seen many amalgam fillings in the years I have been in practice, but have not seen one that did not leak. I do not mean to say that the leaking was such as to do a great amount of harm. It is wonderful how long they preserve teeth and yet are not perfect. I have not seen a really perfect amalgam filling that has been in a tooth for many years.

*Dr. F. J. S. Gorgas.*—By way of relating something that may be interesting in connection with operative dentistry, I will say that many years ago (I think it was more than twenty), shortly after I became connected with the Baltimore College of Dental Surgery, Dr. Clark, of New Orleans, visited that institution and gave a practical illustration of his cylinder-filling method. That method has now, I suppose, become almost obsolete. But there is no question that fillings inserted with cylinders, under favorable conditions, have proven successful in preserving the teeth. I might also, in connection with this subject, refer to a method which perhaps would bring in plastic gold in connection with cylinder filling. Of course at that day we had no such thing as cohesive gold foil. Cohesiveness of foil was considered to be a great objection. These cylinders were made some of hard and some of soft foil. The soft foil was adapted to the walls of the cavity and the hard for the centre. I know of a filling which was inserted in 1851, I think, which, I understood, a few weeks ago was still good.

I still use cylinders in cavities that have firm walls, and I combine with the cylinders plastic gold. After introducing the cylinders, as many as possible in a cavity, and using the soft foil, I affix them firmly to the walls of the cavity and all irregularities of the surface. I then use crystal gold, and insert it by the use of fine-pointed instruments. I think that a very good filling can be obtained in this way. I have been rather successful in using it, but, as I have said, it was made use of under very favorable conditions. Of course I would not recommend such an operation where the walls are unfavorable for it. I merely mention this as a matter of history in connection with the subject of soft and plastic gold.

*Dr. D. Genese.*—In many teeth coming under our notice having gold fillings in them, I find that the crown is perfectly solid, the teeth to all appearances intact, but that the condition of the pulp-cavity shows that the force made use of in inserting that gold has been destructive of the life of the tooth, because it has been excessive; that it has injured the pulp, and that disintegration of the tooth has followed. In the conservation of teeth of a frail character by the method of inserting plastic fillings for a year or so and then removing a portion of the crown of a filling and inserting a harder substance, such as gold or amalgam, I think we can avoid a great deal of the pressure upon the tooth-structure and have a more conservative treatment of them than we have by inserting a gold filling from the foundation of the cavity. In too many instances we adopt a more rapid treatment, on the first visit of our patient, than we are justified in doing. I have observed cases of the insertion of cohesive gold where the operator drilled pits for his starting-point. Many failures occur of the finest gold fillings which were built on this system, resulting in the total death of the pulp. In softer fillings, when they come to the close of the operation, instead of building up cohesive gold on the soft gold, the whole of the approximal cavity is filed away, which not only disfigures the tooth, but, in many instances, leaves a sensitive surface that for many weeks causes the patient great annoyance.

At the cervical border I have seen many failures from the oxy-phosphate and the amalgam not uniting, or from the gold overlapping the edge. In the matter of tin and gold cylinders, too much oxide of tin is deposited, which is so soft that it gives no security against the inroad of fluids destructive to the cavity. Of all the materials, gold is claimed to be the best; but it is the best for grinding surfaces, but not for the approximal surfaces, for unless we get a thorough separation we cannot see our work from all points. The sensitiveness of the teeth, when operating beyond the cervical border, is often in the way of perfect work; but if the separation is maintained and allowed to remain for a day or two, a week, or more after insertion of the filling, so as to enable us to inspect our work when the irritation on the gum has passed away, we shall have a separation which will enable us to control all parts of the work upon which we operate. After the operation on these approximal surfaces I think we are not justified in sending our patient away as if it was wholly finished, but that, in order that we may keep up that separation, we should have the patient visit us again in a day or two, so that we may then inspect the work.

*Dr. E. C. Kirk*, of Philadelphia.—One point in the discussion, mentioned by the gentleman on my left (Dr. Volck), impresses me as one of considerable importance. I refer to what he has said upon the rate of expansion in a gold filling in comparison with the expansion of the tooth in which the filling is inserted. I think that he was hitting very near the truth, although it seems to me that there was one element of error in his position.

If I am not mistaken, Dr. Webb, at one time during his life, undertook an investigation to determine whether the gold which he inserted in the cavity by the mallet was of the same density as the gold theoretically. If I remember correctly his results fell a little short of producing the required density; thus showing that the gold was not absolutely homogeneous. The method which Dr. Volck suggests as having been satisfactory to him in testing this matter seems to me to be open to criticism, because the process which he employs for rolling the gold into plate or drawing it into wire of itself renders the gold thoroughly homogeneous apart from any method which might be employed for inserting it in cavities as a filling.

*Dr. Volck.*—If it be admitted that it has previously been annealed?

*Dr. Kirk.*—Certainly. Dr. Bonwill has brought forward the same experiment to demonstrate that his fillings, inserted by the mallet, were perfectly homogeneous.

The experiment by which Dr. Webb undertook to prove the homogeneity of his fillings, and which I think is the best method that can be employed, is the specific gravity test, if I recollect correctly. I cannot say that I am quoting him literally, but this is my recollection, that the filling inserted by him in a steel plate by means of the electric mallet showed somewhat less specific gravity than that which belonged to pure gold, thus showing possibly that they were not homogeneous. I question very much if we can put in a filling, under any circumstances, which is in a strict sense homogeneous; that is to say, an instance in which the filling expands and contracts as a unit must be a rare one. The point which Dr. Volck has developed in regard to expansion and contraction is certainly a very interesting and important one; but I think that, back of all else, the one primal cause of failure in gold filling at the cervical wall is to be found in the over-malleting of gold at that point. As I have had occasion to state heretofore in dental society meetings when considering the subject, the confusion that exists in the minds of dentists with respect to the terms hard and soft or cohesive and non-cohesive gives rise to this difference of opinion on the subject. There is no reason why a gold foil should not be absolutely soft and absolutely cohesive at the same time. Non-cohesive foil is not necessarily soft. It imparts a feeling of softness to the filling because the lamina of such foil slip or slide upon each other. But if you take an individual sheet, hold it by the corner and shake it, it will in most instances develop that crackling or sharp metallic click indicating hardness. I think that a perfectly accurate adaptation can be had with the strictly cohesive foil which is at the same time soft. Those foils are now made. I think that if we would properly distinguish in the application of the terms which are applied in such cases there would be less confusion and less misunderstanding in our discussions of the subject.

Subject passed.

